# Preparation for the COVID-19 pandemic in the department of radiation oncology in the National Cancer Center Hospital in Tokyo

**DOI:** 10.1093/jrr/rraa031

**Published:** 2020-05-14

**Authors:** Naoya Murakami, Hiroshi Igaki, Hiroyuki Okamoto, Tairo Kashihara, Tomoya Kaneda, Kana Takahashi, Koji Inaba, Kae Okuma, Jun Itami

**Affiliations:** 1 Department of Radiation Oncology, National Cancer Center Hospital, Tokyo, Japan; 2 Department of Medical Physics, National Cancer Center Hospital, Tokyo, Japan

## INTRODUCTION

Since the beginning of the outbreak of novel coronavirus (SARS-CoV-2) disease 2019 (COVID-19) in December 2019 in Wuhan, China [1], the virus has rapidly spread throughout the world. Italy, Spain and the USA suffered the most, and subsequently the World Health Organization declared the COVID-19 outbreak a global pandemic on 12 March 2020. Because Japan is close to China and the Cruise ship ‘Diamond Princess’ arrived at the port of Yokohama as early as 3 February, including passengers infected by the COVID-19, several quarantine measures were put in place without the declaration of an emergency, thus avoiding a negative impact on society and the economy. However, because the number of infected patients whose route of infection was unknown increased, our Prime Minister finally declared a state of emergency on 9 April.

Several organizations or radiotherapy departments from severely affected regions published emergency guidelines or suggested solutions for the management of cancer patients during the global COVID-19 pandemic [2–9].

Under such circumstances, our department also faces a serious situation with regard to how we should prepare for the struggle with this worldwide problem while maintaining the necessary care for cancer patients who need radiation therapy as much as ever. The following is a short summary of our department’s strategy of management for cancer patients who need radiation therapy during the COVID-19 emergency.

## STRATEGY FOR THE MANAGEMENT OF CANCER PATIENTS DURING THE COVID-19 EMERGENCY

The status of the severity of the COVID-19 infection is categorized into four levels according to the number of moderately or critically ill patients in Tokyo (Table 1). Our hospital is not a general hospital, but a national cancer center dedicated to cancer care, management, new treatment development and research. However, if the severity of the COVID-19 infection reaches Level IV, our hospital must receive patients who are infected by COVID-19. The strategy of cancer care in our hospital according to the different phases of COVID-19 is summarized in Table 2. Tokyo declared a state of emergency on 9 April and the current status (when this letter was submitted to the journal) is Phase 1. When the severity of COVID-19 exceeds Level IV, our hospital will start admitting infected patients (Phase 2). Our hospital has 15 hospital wards and each hospital ward has 35–40 beds. As the number of hospital wards involved in the care of infected patients increases, the Phase will rise above 2 (Table 2). As summarized in Table 2, surgery and radiotherapy will be limited according to the severity of the Phase. When the status becomes severer than Phase 2, the number of patients will be reduced according to the priority of radiotherapy summarized in Table 3. In this stage, postoperative adjuvant radiotherapy will be postponed until the Phase becomes better than 1. Primary radiotherapy for diseases in which the efficacy of radiation therapy is proven and the speed of disease progression is not slow, such as head and neck cancer, esophageal cancer, lung cancer and uterine cervical cancer, has high priority. In addition, palliative radiotherapy to alleviate spinal cord compression, bleeding from the tumor or symptomatic brain metastasis also has high priority and treatment should be carried out. Diseases or clinical situations shown as Priority 1 are considered to be top priority, followed by Priority 2 and 3. Radiotherapy for patients who are SARS-CoV-2-positive is postponed until they are proven to be negative, even without respiratory symptoms. Radiotherapy for patients with respiratory symptoms and fever is also postponed until symptom relief occurs, even without SARS-CoV-2 positivity. Suspicious patients are screened for major symptoms (body temperature >37.5°C and respiratory symptoms such as cough, sputum or dyspnea) or past history of having contact with COVID-19 patients within 2 weeks. If SARS-CoV-2 infection is highly suspected, polymerase chain reaction (PCR) testing will be performed. In addition, the following measures are taken to protect medical staff.

- Pregnant physicians or physicians with co-morbidity are asked to avoid the outpatient clinic.
- Use of a flexible laryngofiberscope should be limited unless necessary.
- Personal protective equipment (PPE) such as medical masks are used throughout the day by the all medical staff (standard precaution). If a patient suspected of having SARS-CoV-2 infection attends, medical staff taking care of this patient require further PPE such as gloves, an N95 mask, goggles and a gown.

**Table 1:** Definition of level of the severity of COVID-19 infection

	No. of moderately ill patients in Tokyo	No. of critically ill patients in Tokyo
Level I	<150	<50
Level II	>150	>50
Level III	>450	>100
Level IV	>750	>200
Level V	>1000	>300

**Table 2:** Strategy for cancer care in the National Cancer Center Hospital according to the different phases of COVID-19 severity

Phase		Surgery	Radiotherapy
Phase 0	As usual	As usual	As usual
Phase 1	Under declaration of an emergency	Reduce the number of surgeries by 20%	Reduce fractionation when possible
Phase 2	One ward will be used for COVID-19 patients	Perform only sub-emergency or emergency surgery	Limit to primary and urgent palliative radiotherapy according to the priority summarized in Table 3
Phase 3	Two wards will be used for COVID-19 patients	Perform only emergency surgery	Limit to primary and urgent palliative radiotherapy according to the priority summarized in Table 3
Phase 4	Three wards will be used for COVID-19 patients	Perform only emergency surgery	Limit to primary and urgent palliative radiotherapy according to the priority summarized in Table 3

**Table 3:** Priority of radiotherapy.

Priority	Primary radiotherapy	Palliative radiotherapy
Priority 1	Head and neck cancer, esophageal cancer, lung cancer and cervical cancer	Spinal metastasis with cord compression, bleeding and symptomatic brain metastasis
Priority 2	Primary radiotherapy for rare cancers such as sarcoma, neuroendcrine tumor and malignant melanoma	Painful bone metastasis that cannot be controlled by drugs and supravena cava syndrome for which stent is not indicated
Priority 3	Primary radiotherapy for brain tumors, pancreatic cancer and malignant lymphoma	-

Radiotherapy for patients who are SARS-CoV-2-positive is postponed until they test negative even without respiratory symptoms.

## DISCUSSION

Our strategy for the management of cancer patients during the COVID-19 emergency is briefly summarized in this article. At the time of writing this article, the Phase is 1, however, if the number of moderately or critically ill patients in Tokyo increases further, our hospital must receive infected patients and limit the number of patients who need radiotherapy.

It is anticipated that COVID-19 will spread further outside of Tokyo, therefore, the preparation for the COVID-19 emergency described above will be useful for those regions where the number of infected patients is still low.

The authors strong hope is that after the declaration of an emergency the number of infected patients will be decreased and we can maintain radiation therapy for cancer patients.
